# Bilingual term alignment from comparable corpora in English discharge summary and Chinese discharge summary

**DOI:** 10.1186/s12859-015-0606-0

**Published:** 2015-05-09

**Authors:** Yan Xu, Luoxin Chen, Junsheng Wei, Sophia Ananiadou, Yubo Fan, Yi Qian, Eric I-Chao Chang, Junichi Tsujii

**Affiliations:** 1 State Key Laboratory of Software Development Environment, Key Laboratory of Biomechanics and Mechanobiology of Ministry of Education, Beihang University, Beijing, China; 2 Microsoft Research Asia, Beijing, China; 3 The National Centre for Text Mining, School of Computer Science, The University of Manchester, Manchester, UK; 4 Jinhua People’s Hospital, Jinhua, China

## Abstract

**Background:**

Electronic medical record (EMR) systems have become widely used throughout the world to improve the quality of healthcare and the efficiency of hospital services. A bilingual medical lexicon of Chinese and English is needed to meet the demand for the multi-lingual and multi-national treatment. We make efforts to extract a bilingual lexicon from English and Chinese discharge summaries with a small seed lexicon. The lexical terms can be classified into two categories: single-word terms (SWTs) and multi-word terms (MWTs). For SWTs, we use a label propagation (LP; context-based) method to extract candidates of translation pairs. For MWTs, which are pervasive in the medical domain, we propose a term alignment method, which firstly obtains translation candidates for each component word of a Chinese MWT, and then generates their combinations, from which the system selects a set of plausible translation candidates.

**Results:**

We compare our LP method with a baseline method based on simple context-similarity. The LP based method outperforms the baseline with the accuracies: 4.44% Acc1, 24.44% Acc10, and 62.22% Acc100, where AccN means the top N accuracy. The accuracy of the LP method drops to 5.41% Acc10 and 8.11% Acc20 for MWTs. Our experiments show that the method based on term alignment improves the performance for MWTs to 16.22% Acc10 and 27.03% Acc20.

**Conclusions:**

We constructed a framework for building an English-Chinese term dictionary from discharge summaries in the two languages. Our experiments have shown that the LP-based method augmented with the term alignment method will contribute to reduction of manual work required to compile a bilingual sydictionary of clinical terms.

## Background

Electronic medical record (EMR) systems have become widely used throughout the world to improve the quality of healthcare and the efficiency of hospital services. Accordingly, demands for medical language processing are increasing in order to exploit information found in the text of EMRs. Unlike scientific publications in the medical domain, EMRs are predominantly produced in local languages. To exchange information contained within EMRs across different regions of the world requires the application of multilingual language processing, in which bilingual (or multilingual) lexicons are indispensable resources.

However, a bilingual lexicon of terms used in EMRs is difficult to obtain. Although substantial efforts have been made to manually construct multilingual lexicons based on standard resources in English (UMLS [1], MeSH [2], etc.), health professionals tend to use informal or abbreviated terms in EMRs, most of which are not included in standard resources. A (semi-) automatic method of linking terms from EMRs written in local languages with English terms is a required basic technology to facilitate accurate medical language processing.

In the community of natural language processing (NLP), significant efforts have been made towards the automatic compilation of bilingual lexica. However, straightforward application of the standard methods would not work for terms encountered in EMRs, due to the highly specific nature of the language used in EMRs. A large number of previous efforts have focused on effective use of parallel corpora [3-5], which are not available for EMRs. Several methods have used comparable corpora [6-10], but the nature of the documents contained within them (e.g., patent application documents, newspaper articles in different languages, web pages on the same topics, etc.) generally makes them far more “comparable” than EMRs in different languages. This is because EMRs in different languages follow varying conventions that are specific to their local communities. Discharge summaries in Chinese, for example, are very different from English summaries in their styles. Such characteristics mean than treating collections of EMRs in different languages as comparable corpora requires special treatment.

Furthermore, previous studies in the NLP community have considered only single words, and have not paid enough attention to multi-word terms. While single word terms (SWTs) appear in the clinical domain, the majority of terms in discharge summaries are multi-word terms (MWTs).

In this paper, we adopt a graph-based label propagation (LP) method for generating English translation candidates for SWTs in Chinese summaries. The method was recently proposed for bilingual lexicon compilation using NLP techniques [7]. The method is effective for resolving the difficulties caused by the scarcity of seed words and the discrepancy of context scopes in different languages, which we encounter in comparable corpora of EMRs. Consequently, we expected to improve the performance of work between English and Chinese by applying that approach. With proper selection of seed words and vector representation of context, our experiment shows that the method works significantly better for SWTs in discharge summaries than a baseline method. For MWTs, we develop an alignment method, which uses the results obtained by the LP method to generate translation candidates, and it works much more efficiently than simple LP. Our system shows an improvement in SWTs and proposes a solution for MWTs.

The contributions of this paper are as followed. Our work is the first attempt of English-Chinese lexicon compilation in a specialist domain. Not only the two languages belong to very distant language families but also the comparability of EMR reports in the two languages is very low. Despite these challenges, we show that the context-based approach works reasonably well to generate candidate translations of terms. In particular, we show that the LP method alleviates the difficulty causes by the low comparability of comparable corpus. By integrating the work with a system which facilitates easy human interaction, we expect our work will reduce the human cost of compiling bi-lingual lexicon. The work will also contribute to improving the accuracy and effectiveness of cross-language information retrieval (CLIR) by using sets of candidate translations as synonym terms in retrieval of documents in the target language.

### Related work

Previous approaches to bilingual lexicon compilation can be coarsely classified to context-based methods and those that exploit the internal structure of terms across languages. The latter has been proven to be effective for language pairs which share the same etymology and use similar character sets. It remains a challenge how to exploit the internal structures of terms for a language pair such as Chinese and English. The method proposed in this paper belongs to the former group, which can be further divided into two sub-groups. One sub-group assumes that a parallel corpus exists, while the other only assumes existence of “comparable” corpora. A parallel corpus means a collection of pairs of sentences in two languages which are created by human translators. However, parallel corpora required for bilingual lexicon compilation require translation of large collections of text by human translators, and the construction of such parallel corpora in a specific domain such as the EMR domain would be prohibitively expensive. Therefore, we focus on the use of comparable corpora which are readily available without any involvement of human translation.

Rapp was the first to introduce a context based method using comparable corpora [6]. He applied his method to large comparable corpora of newspaper articles of German and English (135 million words for German and 163 million words for English). He reported an impressive performance of 89% ACC_10_ (top 10 accuracy). However, all of the 100 test words chosen are SWTs and common words with high frequency such as Baby (baby), Brot (bread), Frau (woman), etc.

Subsequent works have shown that the performance of a system is highly dependent on the size and the “comparableness” of the given corpora, language pairs, the availability of seed words, and the frequency of the test words in the corpora. The system developed by Morin et al., which used corpora of web documents in French and Japanese on diabetes and obesity, showed much lower performance, e.g., 51% Acc_20_ for SWTs, and 25% Acc_20_ for MWTs [8]. They used much smaller corpora of 1.5 million-words, although the topics of documents (i.e., diabetes and obesity) were highly restricted. Although their objective is similar to ours (i.e., compilation of a bilingual lexicon for medical terms), the test terms (100 SWTs and 60 MWTs) chosen are terms contained within standard resources such as the ULMS and *Grand dictionnaire terminologique* for French. They used existing bilingual dictionaries of words in the general domain to construct a dictionary of seed words (173,156 entries). Since the corpora which they used (i.e., web documents) contain many words in the general domain, the large set of seed words was found to be effective. Compared with web documents, discharge summaries in Chinese and English contain far fewer general words and far more informal clinical terms. Discharge summaries of the two languages also seem less comparable than web documents (see the Method section for further details). These factors make our task more challenging than theirs.

Given specific comparable corpora, a collection of previous studies have worked on how to define the semantic space of context and how to refine it. Ismail presented a method of identifying the most important contextually relevant seeds to filter out noisy seeds [9]. Graph-based algorithms have also been used for various purposes. Florian and Lukas used a graph to represent linguistic relations (e.g. adjectival modification, subject-verb, etc.) between seed words and the test word (i.e., the word which is to be linked with a translation word in the target language). They proposed a multi-edge extraction method to compute the similarity matrix of all edges [10]. Based on the idea of the SimRank graph similarity algorithm that similar vertices have similar neighborhoods [11], bilingual lexical pairs were extracted by calculating the similarity of vertices across two graphs of the source and target languages.

The work most relevant to ours is Tamura et .al. [7]. They proposed a graph-based label propagation method. The method addresses the difficulties caused by *the scarcity of seed words* and *the discrepancy of context scopes in different languages* which we encounter in discharge summaries. It uses the label propagation algorithms described by Zhu [12] to propagate the context vectors (labels) among seed and test words through a graph of word co-occurrences. The method exploits not only direct contexts but also indirect contexts to form context vectors of test words. Experiments showed that the method consistently outperforms methods using only direct contexts.

Another strand of research relevant to our work is “named entity translation”, which combines named entity recognition (NER) with bi-lingual lexicon compilation [13,14]. This research focuses on the translation of terms (rather than words), which belong to specific semantic types and exploit features specific to those semantic types to extract bilingual lexical pairs. You and Cha used a classifier to distinguish temporal and atemporal entities, and then aligned them in separate procedures [13]. Their method achieved encouraging results in extracting bilingual pairs of person entities from English and Chinese comparable corpora.

## Methods

### Context-similarity-based extraction method – a baseline system

Since the work of Rapp, the framework of context-similarity-based extraction has been used as the core framework in many studies into bilingual lexicon extraction. We use a system based on this framework as a baseline system. The framework extracts a translation pair based on contextual similarity [15], by assuming that a word and its translation appear in similar contexts. The context of a word is represented by a vector, each dimension of which corresponds to a co-occurring seed word in a predefined context window. Since a seed word is paired with its translation in the other language, context vectors of words in the two languages belong to the same vector space. That is, regardless of whether a word belongs to the source or target language, the context vector of the word is represented as

1 $$ \overrightarrow{f}=\left\{{v}_1,{v}_2,\cdots {v}_n\right\} $$

where each dimension corresponds to a word-pair in the seed dictionary. *n* is the size of the seed dictionary. *v*_*i*_, *i* = 1, 2, ⋯ *n* is the weight of word-pair *i* in a vector. A variety of definitions of context have been proposed in previous research, such as: a predefined window [6], a sentence [16], a paragraph [17], and predecessors and successors in dependency parse trees [18]. Weights used in previous work are also varied, such as frequency, tf-idf [19], log-likelihood [6], etc.

Once words in the source and the target languages are represented by context vectors of seeds, we can compute context similarities between words in the two languages by the cosine similarity of two vectors, that is,

2 $$ {w}_{ij}=Cos\left(\overrightarrow{f_i},\overrightarrow{f_j}\right)=\frac{\overrightarrow{f_i}\cdot \overrightarrow{f_j}}{\left\Vert \overrightarrow{f_i}\right\Vert \left\Vert \overrightarrow{f_j}\right\Vert }. $$

According to their cosine similarities with the test word, we can rank the translation candidates in the target language.

Based on reasons discussed in the next section, we use specialised terms (i.e., named entities) as seeds, instead of words. This means that our work diverges from previous efforts in the NLP community. In the same way as research into named entity translation, our test words are actually terms, not words. Hence, we use the terms *seed terms* and *test terms* in the description of our approach, instead of seed words and test words.

In order to avoid the difficulty caused by discrepancy of context units in the two languages (see the following section), the baseline system uses a narrow window of context, that is, two seed terms, one before the test term and another after the test term. For weights in a vector, the baseline system uses the frequency.

### Discharge summaries as comparable corpora and the core framework

Discharge summaries of Chinese and English are quite different from and less comparable than other comparable corpora used by the NLP community, and thus require special treatment.

First of all, sentences in Chinese summaries tend to be much longer than those in English summaries. In Chinese summaries, health professionals enumerate the patient’s chief complaints, diagnostic findings and therapies administered in very long sentences. Sometimes an entire paragraph consists of only one sentence.

**Figure 1:**
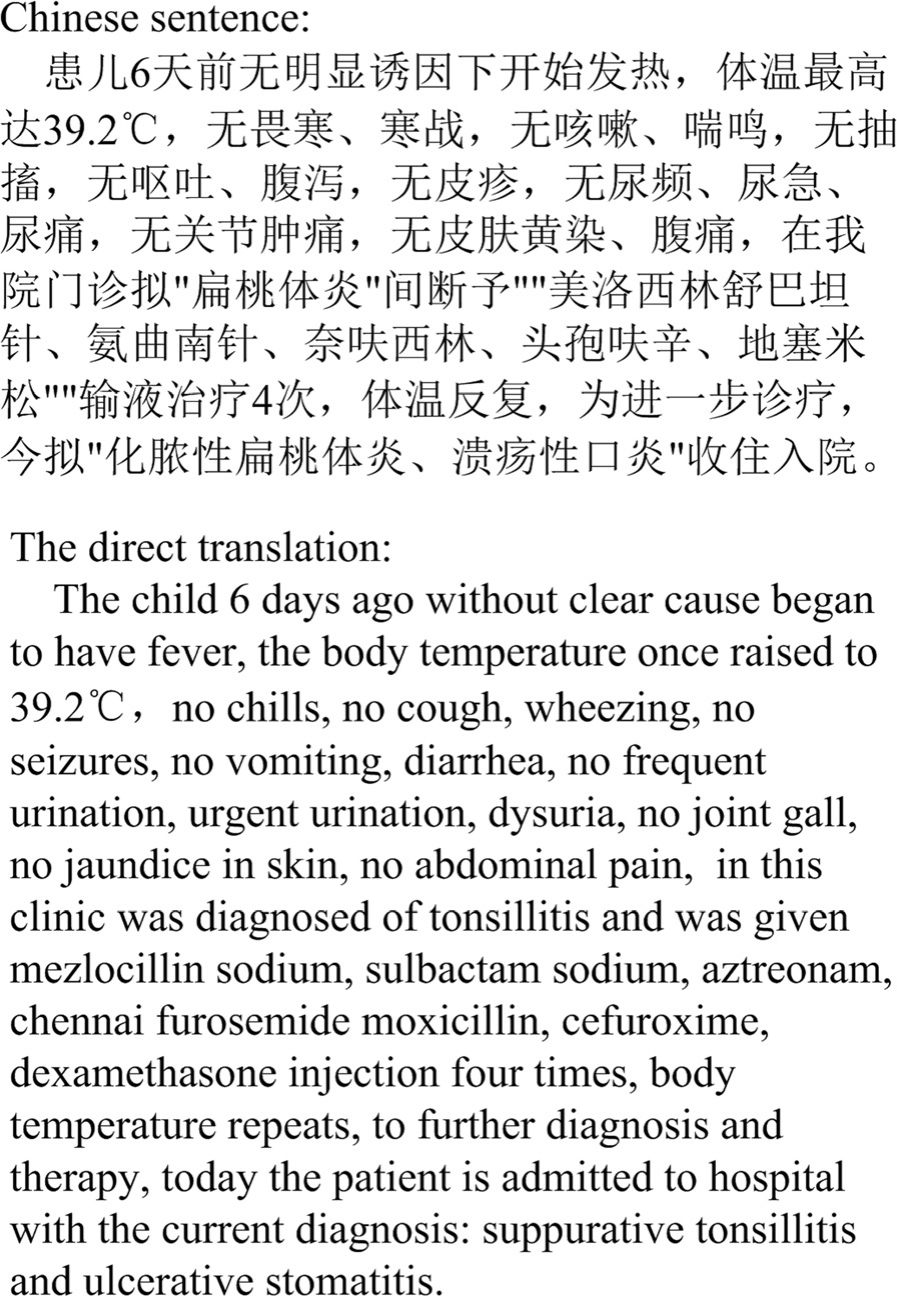
A sentence in a Chinese discharge summary.

Figure 1 shows a typical “sentence” in a Chinese summary. It contains a long list of itemized without-phrases (phrases following the preposition of 无 – “without” in Chinese). Although the direct translation in English (Figure 1) is understandable, such a sentence rarely appears in English summaries. In the i2b2 collection of English summaries [20], itemized lists appear only in descriptions of medications and their dosages, or medical tests and their results. Furthermore, only a very restricted set of general language words (e.g., 明显 (obvious), 出现 (appear), etc.) appears in summaries, particularly in Chinese ones.

In short, while English summaries constitute narrative texts and consist of normal sentences, occasionally intertwined with shorter sentences and itemizations, Chinese summaries are more like memos, generally containing sequences of specialist terms juxtaposed by a small set of general words (e.g., prepositions, verbs, etc.).

Based on these characteristic differences in style, we decided to use only specialist terms (i.e. named entities), rather than general words, as seeds. We use 1,200 seeds terms, which are contained within in a domain-specific bilingual lexical resource, the Chinese Medical Subject Headings (CMeSH) [2], and which appear in our comparable corpora. Note that the number of seed terms and thus the dimension of a vector are much smaller than those usually used by the NLP community.

As in the framework of “named entity translation”, Chinese named entity recognizers (NERs) [21] play an important role in our system. They recognize test terms (i.e., Chinese terms which are terms not in the seed set and should be linked with English terms) as well as the seed terms in their contexts.

Furthermore, since a “sentence” in a Chinese summary tends to be much longer than in an English summary, using the unit of a sentence or a larger unit, such as a paragraph, as context leads to large discrepancy in the scope of context between the two languages. Such a discrepancy in context skews the contextual similarity between words in the two languages. To avoid such problems, we use a narrow window of context e.g. two neighboring seed terms.

The narrow scope of context and the small dimension of the vector space generally have adverse effects on the performance of a context-similarity-based extraction system. In order to alleviate these problems, we adopt the graph-based label propagation (LP) framework [7], as our core framework. The framework enriches context vectors by using indirect contexts and can expand the scope of context dynamically.

### Processing flow of the proposed system

**Figure 2:**
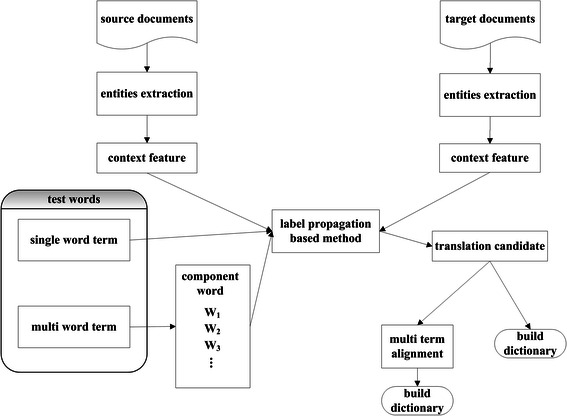
Construction of bilingual lexicons from discharge summaries in English and Chinese.

Figure 2 shows the flow of processing in our system. Before bilingual lexicon extraction, we first apply NERs [21,22] to discharge summaries in the two languages. These two monolingual processing phases produce a set of test terms (i.e., terms not appearing in the seed dictionary) to be linked, and locate where the predefined 1,200 seed terms appear.

Bilingual lexicon compilation is performed in two stages, the first dealing with SWTs, for which the LP framework is used, and the second for MWTs, which is based on alignment of component words. Below, we describe these two phases in detail.

### LP framework for SWTs

In a similar way to the context-similarity based framework, the LP framework, which was proposed by Tamura et al. [7], produces vector representations of words in the two languages. Since each dimension of the vector space corresponds to a seed term, in the same way as in the context-similarity framework, the similarity of two words in the source and target languages can be computed, and plausible translation candidates can be chosen. The major difference between the two frameworks is in the how vector representation of a word is produced. Instead of using a fixed window of context, the LP framework uses an iterative process of propagating vector representations through a graph. In the graph, there are two types of vertices, i.e., labelled ones, which correspond to seed words, and unlabelled ones, which correspond all other words. The following is the original procedure proposed by Tamura et al.:

1. Graph construction

Let *V* = {*v*_1_, ⋯, *v*_*n*_} be a set of vertices of a graph, where *v*_*i*_ represents a word from a corpus. Let *E* and *W* be a set of edges and the corresponding weights. *e*_*ij*_ ∈ *E* denotes the edge between *v*_*i*_ and *v*_*j*_, whose weight is *w*_*ij*_ ∈ *W*. The weight, *w*_*ij*_, is meant to express the strength of the connection of the two vertices (i.e. words).

2. Label propagation

Label propagation (LP) is an iterative algorithm, which propagates labels (i.e. vector representations) from labelled vertices to unlabelled vertices to infer the labels of unlabeled vertices. During the propagation, a vertex’s label propagates to neighbouring vertices according to the edge weights. Finally, vertices with similar neighbours will have similar labels.

The initial vector representation attached to a vertex is defined as follows:

3 $$ {q}_i^0(z)=\left\{\begin{array}{cc}\hfill 1\hfill & \hfill if\kern0.5em {v}_i\in {V}_s\kern0.5em  and\kern0.5em z={v}_i\hfill \\ {}\hfill 0\hfill & \hfill if\kern0.5em {v}_i\in {V}_s\kern0.5em  and\kern0.5em z\ne {v}_i\hfill \\ {}\hfill u(z)\hfill & \hfill otherwise\hfill \end{array}\right. $$

Where ${q}_i^k=\left(i=1\cdots \left|V\right|\right)$ is the vector representation of *v*_*i*_ after *k* cycles of propagation. ${q}_i^k(z)$ is the value of the dimension corresponding to the seed word z. _*Vs*_ is the set of seeds. *u*(*z*) is the value of label *z*in a uniform distribution

The vector representation of each vertex is updated in each cycle of iteration as follows:

4 $$ {q}_i^m(z)=\left\{\begin{array}{c}\hfill {q}_i^0(z)\kern6.2em  if\kern0.5em {v}_i\in {V}_s\hfill \\ {}\hfill \frac{{\displaystyle {\sum}_{v_j\in N\left({v}_i\right)}{w}_{ij}\cdot {q}_j^{m-1}(z)}}{{\displaystyle {\sum}_{v_j\in N\left({v}_i\right)}{w}_{ij}}} otherwise\hfill \end{array}\right. $$

Larger edge weights allow easier propagation of labels. After several iterations, ${q}_i^k(z)$ of all vertices will converge to stable values. We ran this procedure for 10 iterations in our experiment.

There are two obvious alternatives to calculating the weight, *w*_*ij*._ One alternative would be to use co-occurrence frequency of the two words within a fixed context window. The other alternative is to use the context-similarity used in the baseline system. Our findings about these two alternatives agree with those of Tamura et. al. i.e., the context similarity performs better than the co-occurrence frequency. Accordingly, we use context similarity as the weight in our experiments.

Since we use specialist terms (named entities) instead of words, vertices in the graph correspond to terms, the majority of which are non-labelled (non-seed terms). In the context-similarity framework, non-seed terms play no role in the vector representation of a given term, even if they occur in the neighbourhood of the term. On the other hand, in the LP framework, they play the significant role of intermediate vertices through which labels are propagated. This mitigates adverse effect of a small number of seed terms. Furthermore, each cycle of iteration expands the context to be considered, by including information from more remote vertices. This dynamic expansion of context resolves the difficulty caused by the discrepancy of scopes of context in the two languages, which is encountered in the approach based on context-similarity with a fixed context unit.

### Alignment of multi-word term

The majority of the methods developed by the NLP community consider only SWTs. However, many MWTs appear in discharge summaries, which is one of the major causes of poor performance. Even when the component words appear frequently individually, MWTs that combine them tend to have much lower frequency in a corpus. The performance of a system is usually much worse for words/terms with low frequency in the corpus on which the system is trained.

A straightforward solution is to use the principle of compositionality, that is, the “translation of a whole can be compositionally computed from translations of its parts” [22]. This principle works for pairs such as “腰椎 穿刺” - “lumbar puncture”. 腰椎and穿刺are translated into “lumbar” and “puncture”, respectively. However, the principle is violated in many cases. The Chinese term “吞咽 困难”, for example, can be translated compositionally into “swallow difficulty”, but in English summaries, the same concept appears as “dysphasia”. Terms in different languages are not always translated compositionally. This is particularly true for a language pair such as Chinese-English, since the languages do not share the same etymology or character sets.

In order to account for the above, we account for all the possibilities that a term of *m* words may be translated into an *n* word-length term. We call this an “*m*-to-*n*” pair. For example, the Chinese SWT, “心衰”, is translated into ”heart failure” (1-to-2 pair), while the Chinese MWT, ”甲状腺 功能 减退”, is translated into “hypothyroidism” (3-to-1 pair). The three component words in Chinese for “hypothyroidism” mean “thyroid gland”, “function” and “degradation”.

**Figure 3:**
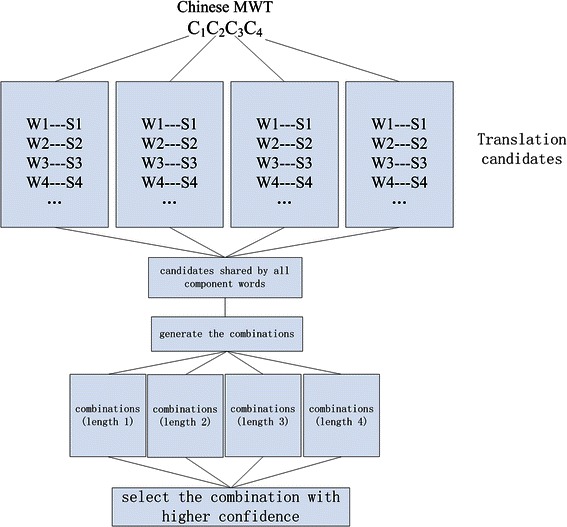
Multi term alignment figure legend text.

In the processing flow of our system, Chinese terms are first recognized by an NER based on a joint model of NER and word segmentation [21]. The NER produces not only boundaries of terms but also segmentation of sentences into words. If a recognized term contains more than one word in its scope, the term is treated as an MWT and its translation candidates are produced according to the following steps (see Figure 3):

**Step1:** Generate a set of translation candidates in English for each of the component words by the LP method. This is done by constructing a graph in which the component words are treated as separate vertices.

**Step2**: Create a set of translation candidates, which is the intersection of the sets created in **Step1.**

**Step3:** For each of the subsets of *j* words (1 ≤ *j* ≤ *n*) in the set created in **Step2**, check whether a term containing all of the words in the subset (but no other words) appears in one or more of the English summaries. If such an English term appears, add it to the set of candidate translations of the MWT.

**Step4**: Calculate the translation plausibility of each candidate in the set created in **Step3**, and order them accordingly.

We observe that, since the component words of an MWT usually appear in similar contexts, they share many translation candidates. A component word of the correct translation for the MWT is normally among these shared candidates. **Step 2** is based on this observation. Table 1 shows concrete examples of this observation for a 2–2 pair and a 2–1 pair.

**Table 1:** **T** **ranslation Candidates for “心房”, “颤动”, “吞咽”, and “困难”**

**心房**	**颤动**	**吞咽**	**困难**
Bradycardia	bradycardia	hematuria	hematuria
**fibrillation**	**fibrillation**	**dysphagia**	syncope
acidosis	acidosis	syncope	**dysphagia**
angina	angina	stools	fever
depression	rheumatoid	fever	stools
hypothyroidism	myocardial	acidosis	jaundice
syncope	pericarditis	bloody	anemia
encephalopathy	cesarean	jaundice	acidosis
heart	heart	anemia	sepsis
rheumatoid	leukemia	angina	bloody
glaucoma	glaucoma	sepsis	respiratory
dysphagia	cardiomyopathy	thrombocytopenia	angina
**atrial**	encephalopathy	respiratory	encephalopathy
thrombocytopenia	hypothyroidism	bronchitis	leukemia
cardiomyopathy	syncope	bacteremia	thrombocytopenia
cesarean	palsy	leukemia	bacteremia
anemia	**atrial**	fibrillation	fibrillation

Bold word means the correct translation (“atrial fibrillation” for “心房颤动” and “dysphagia” for “吞咽困难”).

In the 2–2 pair of “心房 颤动” –“atrial fibrillation”, each of the two component words in English translation, “atrial” and “fibrillation”, appear as highly ranked candidates for the Chinese component words of “心房” and “颤动”. On the other hand, in the example of a 2–1 pair, “吞咽 困难”-“dyshapia”, the correct translation, “dyshapia” appears as a highly ranked candidate for both of the Chinese component words.

**Step3** allows all the possibilities of *m*-*n* pairs to be considered (it checks $\frac{q-{q}^n}{1-q}$ subsets where *q* is the number of candidates in the intersection set and *n* is the maximum number of the component words in English). Although the number of the subsets to be considered is large, only a small fraction of them actually appears in English summaries, and so most of them are discarded as translation candidates. On average, we generate 8000 subsets but only 220 appear as English terms in English summaries.

We use a rather simple plausibility measure for ranking, i.e., the average of the similarity values between component words, as calculated when extracting translation pairs for SWTs.

## Results

### Experiment data

We used the dataset of 2012 i2b2 challenge [20] for English discharge summaries. Chinese discharge summaries are provided by the Jinhua People’s Hospital in Zhejiang province, China. The datasets contain 100,745 and 62,231 summaries, respectively. The NERs are applied to extract named entities (i.e., terms) from the summaries. We use our own English and Chinese NERs, both of which show the state of the art performance [21,23]. For the purposes of our experiments, we retain only those terms belonging to the categories *Medical Problem*, *Treatment* and *Test*. This results in 31,692 terms in English summaries and 8,579 terms in Chinese summaries.

We extracted a set of seed terms from CMeSH [2]. The number of terms in CMeSH is 48,442. The set of seed terms contain 1,220 seeds, all of which are SWTs. Although CMeSH is a large standard dictionary for the biomedical domain, only 1,220 of the terms contained within it occur in both the Chinese and English corpora. This shows the discrepancy between standard terms and terms actually used in EMRs. Compared with the number of seeds used for other comparable corpora such as newspaper articles, patent documents, web pages, etc., the set of seeds we can use for EMRs is much smaller.

We manually selected 100 Chinese terms as our test data, which consist of 37 MWTs and 63 SWTs. They were not covered by the seed dictionary. The 37 Chinese MWTs are manually translated. The distribution of *n-m* pairs in MWTs is shown in Table 2. Note that more than half of MWTs do not follow compositional translation.

**Table 2:** **The lengths of 37 multi word translation pairs**

***m*** **to** ***n*** **pair**	**Number**
2 to 1	11
2 to 2	14
3 to 2	2
3 to 3	1
1 to 2	7
2 to 3	2

### Experimental results

In our framework, all the component words in Chinese MWTs were treated as SWTs in Step 1 of the MWT translation process. From 37 MWTs, we get 72 SWTs. We use these SWTs, together with the other 63 SWTs as the test set for the LP method. Note that for some of the test terms (around 10), their translation pairs may not be present in the English corpus. We evaluated the LP based method against the baseline system using the context-similarity-based method.

Table 3 shows the performance of the two systems in terms of Top N accuracy. The result shows that the LP based methods outperform the baseline system by a large margin. The improvements are statistically significant for Acc10 and Acc100.

**Table 3:** **Performance on SWTs translation**

	**Acc**_**1**_	**Acc**_**10**_	**Acc**_**100**_
LP	4.44%	24.44%	62.22%
Baseline	3.70%	8.89%	20.74%

Table 4 shows the results of our experiment with the 37 MWTs. The baseline method treats the MWTs as SWTs using the LP based method. The accuracy of our system, which uses the word alignment, is far superior to the simple LP method. The improvements are all more than 10% for Acc10, Acc20 and Acc50.

**Table 4:** **Performance on MWTs translation**

	**Acc**_**10**_	**Acc**_**20**_	**Acc**_**50**_
Multi term alignment	16.22%	27.03%	29.73%
LP	5.41%	8.11%	16.22%

## Discussion

The Top N accuracy figures of our system, which extracts bilingual medical lexicon from medical discharge summaries, are lower than those reported by the other studies in the NLP community, concerning different corpora. However, it is simply not possible to make comparisons between the Top N accuracy figures of systems with diverse characteristics. The performance of a system is highly dependent on the size and the “comparableness” of given corpora, language pairs, the availability of seed words, and the frequency of test words in corpora. The task approached in this paper is one of the toughest tasks in every aspect of these factors. Though comparable in a broad sense, the English and Chinese summaries which we used are far less comparable than those typically used by the NLP community. Furthermore, the two languages involved, i.e., English and Chinese, do not share the same etymology and character sets, which makes many clues used in bilingual lexicon compilation by the NLP community infeasible and makes translation of MWTs more difficult than other pairs of languages.

The results of our experiments show that the LP-based and word alignment method contribute to resolving the difficulties caused by the scarcity of seeds, the discrepancy of context in the two languages, and the low frequency of MWTs in the corpora. We have shown that these two methods significantly outperform the baseline systems.

It should also be noted also that our evaluation criteria may sometimes be too strict, since we do not consider that a permissible translation is a correct translation. For example, an accurate translation of the Chinese SWT “虹膜” (irides) was not found, while the English word “iridectomy” ranks in 82nd position. In the context of SWT translation, we do not treat such permissible or relevant translation terms as correct ones. In the translation of MWTs such as “虹膜 切除”(“irides excision”), however, all translation candidates of SWT “虹膜” are considered as potentially correct translations for equivalent English MWTs.

We have shown that the multi term alignment method can improve the performance when building lexicons for MWTs. However, when generating the combinations from the translation candidates, the length of the correct translation is uncertain. Therefore, we assume that the length of the correct translation is less than the length of the MWT in the source language. Therefore, this method cannot find the correct translation of MWTs with a length longer than the MWT in source language. However, as shown in Table 1, 24.32% of the 37 test Chinese MWTs have a longer translation in English than the original MWT.

### Error analysis

Since the current system does not deal with abbreviations, we treat full forms and abbreviated forms of the same terms as distinct terms. Since abbreviations are abundant in discharge summaries, this introduces unnecessary vertices into the graph used by the LP method, together with unnecessary noise. Furthermore, the different character sets of the two languages lead to very different abbreviation conventions. For example, “硬膜外连续麻醉”(continuous peridural anesthesia), is abbreviated as “硬连麻”. The former is treated as an MWT, while the latter as an SWT. No corresponding abbreviations appear in the English summaries. On the other hand, however, abbreviations in English are often ambiguous, e.g., “pt”, which could correspond to “patient” or “physical therapy”. A possible solution would be a context-based one. First, we could get abbreviations and their original words from monolingual Wikipedia. Then, a context-based method could be applied to find which one is the correct original phrase for each abbreviation. Finally, we treat an abbreviation and its original phrase as the same word and then apply our system.

According to efficiency considerations, we retain only the 100 top-ranking translation candidates for each component word in an MWT, which can sometimes cause problems. For example, the translations “pleural” and “effusion” do not appear in the top 100 candidate translations of the component words of “胸腔 积液 (pleural effusion)”. The other main source of errors is that our method cannot translate pairs where the word in the source language is shorter than its translation. For example, the translations of “二尖瓣 置换 (mitral valve replacement)”, “结肠癌 (colon cancer)” cannot be found by our method. For the latter two errors, swapping the position of English and Chinese could be a solution.

## Conclusion

We have developed a system which links terms in Chinese discharge summaries with those in English summaries. The system uses the Label Propagation (LP) method as the core framework and augments it by using terms, rather than words, as the vertices in the graph and as seeds. The method was proven to cope with the difficulties caused by the scarcity of seeds and the discrepancy of context in the two languages much more effectively than the baseline system, which employs the context-similarity framework. We have further improved the system by introducing a new method which treats non-compositional translation of MWTs in Chinese.

Our work still has some potential improvements. The system uses the context-based approach, and does not use any information of the internal structures of terms. Since Chinese and English use completely different set of characters and their terms have completely different etymologies, naïve application of the structure-based approach [24] does not work. However, recent study [25] showed that a refined method of the structure-based approach actually worked for lexicon compilation of English-Japanese, which have the same challenges as English-Chinese. Furthermore, they showed that combination of the two approaches significantly improve the performance. The next step for improving the performance is to adapt a method similar to them.

The cost of human intervention varies significantly, depending on functions which an integrated system provides for human intervention. In order to show the effectiveness of our work, we have to develop a system which includes a component based on our work.

### Availability of supporting data

The data sets supporting the results of this article are available in the [repository name] repository, [unique persistent identifier and hyperlink to datasets in https://drive.google.com/folderview?id=0B7VxMGBuf9HvLTJXaFBUMDE0WWc&usp=sharing].

## Abbreviations

SWTs: Single-word terms
MWTs: Multi-word terms
LP: Label propagation
CMeSH: Chinese Medical Subject Headings
EMR: Electronic medical record
NLP: Natural language processing
NER: Named entity recognition
NERs: Named entity recognizers
CLIR: Cross-language information retrieval

## References

[1] Li D, Hu T, Li J, Qian Q, Zhu W. Construction and application of the Chinese unified medical language system. J Intelligence. 2011;30(2):147–51.

[2] Wang X, Thompson P, Tsujii J, Ananiadou S. Biomedical Chinese-English CLIR using an extended CMeSH resource to expand queries. In: Proceedings of the 18th International Conference on Language Resources and Evaluation; 2012. p. 1148-1155.

[3] Wu D, Xia X. Learning an English-Chinese lexicon from a parallel corpus. In: Proceedings of the 1st Conference of the Association for Machine Translation in the Americas; 1994. p. 206–13.

[4] Fung P. A statistical view on bilingual lexicon extraction: from parallel corpora to non-parallel corpora. In: Proceedings of the 3rd Conference of the Association for Machine Translation in the Americas on Machine Translation and the Information Soup; 1998. p. 1–17.

[5] Fung P. A pattern matching method for finding noun and proper noun translations from noisy parallel corpora. In: Proceedings of the 33rd annual meeting on Association for Computational Linguistics; 1995. p. 236–43.

[6] Rapp R. Automatic identification of word translations from unrelated English and German corpora. In: Proceedings of the 37th annual meeting of the Association for Computational Linguistics on Computational Linguistics; 1999. p. 519–26.

[7] Tamura A, Watanabe T, Sumita E. Bilingual lexicon extraction from comparable corpora using label propagation. In: Proceedings of the 2012 Joint Conference on Empirical Methods in Natural Language Processing and Computational Natural Language Learning; 2012. p. 24–36.

[8] Morin E, Daille B, Takeuchi K, Kageura K. Bilingual terminology mining-using brain, not brawn comparable corpora. In: Proceedings of the 45th Annual Meeting of the Association for Computational Linguistics A; 2007. p. 664-701.

[9] Ismail A, Manandhar S. Bilingual lexicon extraction from comparable corpora using in-domain terms. In: Proceedings of the 23rd International Conference on Computational Linguistics; 2010. p. 481–89.

[10] Laws F, Michelbacher L, Dorow B, Scheible C, Heid U, Schütze H. A linguistically grounded graph model for bilingual lexicon extraction. In: Proceedings of the 23rd International Conference on Computational Linguistics; 2010. p. 614–22.

[11] Jeh G, Widom J. SimRank: A measure of structural-context similarity. In: Proceedings of the 8th ACM SIGKDD international conference on Knowledge discovery and data mining; 2002. p. 538–43.

[12] Zhu X, Ghahramani Z. Learning from labeled and unlabeled data with label propagation. Technical Report CMU-CALD-02-107, Carnegie Mellon University; 2002.

[13] You G, Cha Y, Kim J, Hwang S. Enriching entity translation discovery using selective temporality. In: Proceedings of the 51st annual meeting of the Association for Computational Linguistics on Computational Linguistics; 2013. p. 201-05.

[14] Lee T, Hwang S. Bootstrapping entity translation on weakly comparable corpora. In: Proceedings of the 51st annual meeting of the Association for Computational Linguistics on Computational Linguistics; 2013. p. 631-40.

[15] Andrade D, Nasukawa T, Tsujii J. Robust measurement and comparison of contextsimilarity for finding translation pairs. In: Proceedings of the 23rd International Conference on Computational Linguistics; 2010. p. 19–27

[16] Laroche A, Langlais P. Revisiting context-based projection methods for term-translation spotting in comparable corpora. In: Proceedings of the 23rd international conference on computational linguistics; 2010. p. 617–25.

[17] Fung P, McKeown K. Finding terminology translations from non-parallel corpora. In: Proceedings of the 5th Annual Workshop on Very Large Corpora; 1997. p. 192–202.

[18] Garera N, Callison-Burch C, Yarowsky D. Improving translation lexicon induction from monolingual corpora via dependency contexts and part-of-speech equivalences. In: Proceedings of the 13th Conference on Computational Natural Language Learning; 2009. p. 129–37.

[19] Fung P, Yee L. An IR approach for translating new words from nonparallel, comparable texts. In: Proceedings of the 17th international conference on Computational linguistics; 1998. p. 414–20.

[20] Uzuner O, South B, Shen S, Duvall S. 2010 i2b2/VA challenge on concepts, assertions, and relations in clinical text. Journal of the American Medical Informatics Association. 2011; 18(5):552–6. 10.1136/amiajnl-2011-000203 PMC3168320 21685143

[21] Xu Y, Wang Y, Liu T, Liu J, Fan Y, Qian Y, Tsujii J, Chang E. Joint segmentation and named entity recognition using dual decomposition in Chinese discharge summaries. Journal of the American Medical Informatics Association; 2014; 21(1): 84-92. 10.1136/amiajnl-2013-001806 PMC3957392 23934949

[22] Kaji H, Tsunakawa T, Komatsubara Y. Improving Compositional Translation with Comparable Corpora. In: Proceedings of the 5th Workshop on Building and Using Comparable Corpora; 2012. p. 134–42.

[23] Xu Y, Hong K, Tsujii J, Chang E. Feature engineering combined with machine learning and rule-based methods for structured information extraction from narrative clinical discharge summaries. J Am Med Inform Assoc. 2012; 19(5): 824–32. 10.1136/amiajnl-2011-000776 PMC3422834 22586067

[24] Langlais P, Yvon F, Zweigenbaum P. Improvements in analogical learning: application to translating multi-terms of the medical domain. In: Proceedings of the 12th Conference of the European Chapter of the Association for Computational Linguistics; 2009. p. 487–95

[25] Kontonatsios G, Korkontzelos I, Tsujii J, Ananiadou S. Combining string and context similarity for bilingual term alignment from comparable corpora. In: proceedings of the 14th Conference on Empirical Methods in Natural Language Processing; 2014. p. 1701–12.

